# Dissociation Between Functional Performance and Structural Joint Damage Following Sertraline Treatment in Collagen-Induced Arthritis

**DOI:** 10.3390/ijms27167251

**Published:** 2026-08-14

**Authors:** Grzegorz Chmielewski, Mateusz Mikiewicz, Jakub Kuna, Łukasz Jaśkiewicz, Joanna Czerwińska, Michał Majewski, Magdalena Krajewska-Włodarczyk

**Affiliations:** 1 Department of Rheumatology, School of Medicine, Collegium Medicum, University of Warmia and Mazury in Olsztyn, 10-719 Olsztyn, Poland; kuna.jakub@wp.pl (J.K.); magdalenakw@op.pl (M.K.-W.); 2 Department of Pathological Anatomy, Faculty of Veterinary Medicine, University of Warmia and Mazury in Olsztyn, 10-719 Olsztyn, Poland; mateusz.mikiewicz@uwm.edu.pl; 3 Department of Human Physiology and Pathophysiology, School of Medicine, Collegium Medicum, University of Warmia and Mazury in Olsztyn, 10-719 Olsztyn, Poland; lukasz.jaskiewicz@uwm.edu.pl; 4 Department of Dermatology, Sexually Transmitted Diseases and Clinical Immunology, School of Medicine, Collegium Medicum, University of Warmia and Mazury in Olsztyn, 10-719 Olsztyn, Poland; joanna.czerwinska@uwm.edu.pl; 5 Department of Pharmacology and Toxicology, School of Medicine, Collegium Medicum, University of Warmia and Mazury in Olsztyn, 10-719 Olsztyn, Poland; michal.majewski@uwm.edu.pl

**Keywords:** rheumatoid arthritis, collagen-induced arthritis, sertraline, inflammation

## Abstract

Rheumatoid arthritis is a chronic inflammatory joint disease often accompanied by depressive symptoms, in which functional impairment does not always reflect the extent of structural joint damage. Sertraline, a selective serotonin reuptake inhibitor, may influence behavioural and neuroimmune pathways, but its effects on the relationship between joint pathology and functional performance remain unclear. This study evaluated whether sertraline affects functional performance independently of histopathological joint damage in collagen-induced arthritis. Male Wistar rats with collagen-induced arthritis were assigned to untreated or treatment groups receiving sertraline, methotrexate, combined methotrexate and sertraline, infliximab, or tocilizumab. Functional performance was assessed by spontaneous wheel-running activity, body weight, and joint swelling. At week 12, ankle joints underwent histopathological evaluation, and haematological parameters and serum cytokines were analysed. Sertraline did not reduce synovial inflammation, cartilage damage, or bone erosion, although sertraline-treated rats showed a smaller numerical decline in spontaneous wheel-running activity than rats with untreated collagen-induced arthritis. In contrast, methotrexate and biologic therapies improved both structural damage and functional outcomes. Functional performance did not consistently correlate with histopathological severity or circulating cytokine levels. Sertraline did not produce a significant improvement in histopathological outcomes compared with untreated collagen-induced arthritis, while the smaller numerical decline in exploratory wheel-running activity should be interpreted cautiously.

## 1. Introduction

Rheumatoid arthritis (RA) is a chronic, autoimmune inflammatory joint disease that predominantly affects the small joints and leads to progressive structural damage. Persistent synovial inflammation results in synovial membrane hypertrophy, joint swelling, and pain, ultimately contributing to the destruction of articular cartilage and subchondral bone [1]. Inflammation in RA is systemic in nature; proinflammatory cytokines released into the circulation, such as tumour necrosis factor-α (TNF-α), interleukin-6 (IL-6), and interleukin-1β (IL-1β), contribute to systemic symptoms and may lead to damage in multiple organs [2].

Depression is among the most common comorbidities observed in patients with RA. Its presence markedly reduces the quality of life and decreases the chances of achieving clinical remission. It also impairs adherence to therapy and is linked with higher disease activity, greater disability risk, and increased mortality. Evidence indicates a bidirectional relationship between RA and depression: the chronic pain, disability, and inflammation characteristic of RA predispose patients to depressive symptoms, while depression itself can exacerbate RA activity and symptom severity [3]. Consequently, the prevalence of depression in RA is significantly higher than in the general population, and it is associated with greater pain intensity, fatigue, and physical impairment [4]. A recent study has emphasized the role of inflammation in the pathophysiology of depression. Individuals with depressive disorders frequently exhibit elevated circulating levels of C-reactive protein (CRP), IL-1β, TNF-α, and IL-6 when compared to the general population [5]. In depression, immune system dysregulation affects both innate and adaptive immunity [6]. This imbalance between immune activation and suppression results in impaired cellular immunity and the establishment of chronic low-grade inflammation. Heightened responsiveness to pathogens and stressors triggers a pronounced proinflammatory response, which subsequently induces compensatory anti-inflammatory pathways. In genetically or environmentally predisposed individuals, this persistent immunological imbalance contributes to neuroendocrine axis dysfunction, chronic stress, pain hypersensitivity, anxiety, and depressed mood, ultimately promoting the onset of depressive disorders [7]. In inflammatory arthritis, structural joint damage does not always correlate with functional impairment or patient-reported disability, which may be influenced by systemic inflammation, pain perception, fatigue, and neuroimmune mechanisms [8].

Proinflammatory cytokines have also been implicated in neuroinflammation observed in the brains of individuals with depression. These mediators, reaching the central nervous system either directly or indirectly, disrupt monoaminergic neurotransmitter metabolism and thereby impair psychological and cognitive functioning, contributing to the development of depressive symptoms [3]. Furthermore, cytokine-mediated activation of enzymes involved in serotonin and tryptophan degradation reduces serotonin availability. Concurrently, increased production of N-methyl-D-aspartate (NMDA) receptor agonists within the immune system contributes to glutamate excess. These two mechanisms are strongly implicated in the depression pathogenesis [9].

Sertraline (SRT), a selective serotonin reuptake inhibitor (SSRI), is widely used in the treatment of depression [10]. Although its primary therapeutic action is attributed to modulation of serotonergic neurotransmission, accumulating evidence suggests that sertraline also exerts immunomodulatory effects [11]. The drug reduces concentrations of proinflammatory cytokines, particularly IL-6 and TNF-α, while simultaneously increasing levels of the anti-inflammatory cytokine interleukin 10 (IL-10) [12]. These findings suggest that sertraline may influence inflammatory pathways relevant to both depression and chronic inflammatory diseases such as RA.

Methotrexate (MTX) is an anchor conventional synthetic disease-modifying antirheumatic drug used in the treatment of RA. At the low doses used in inflammatory diseases, its anti-inflammatory activity is thought to involve several mechanisms, including increased extracellular adenosine signalling and modulation of immune-cell activation and cytokine production [13]. Infliximab (IFX) is a chimeric monoclonal antibody that binds soluble and transmembrane TNF-α, thereby inhibiting TNF-mediated inflammatory signalling [14]. Tocilizumab (TCZ) is a humanised monoclonal antibody directed against both soluble and membrane-bound IL-6 receptors, preventing IL-6-mediated signalling [15]. In the present study, these agents were included to provide pharmacological context representing conventional and cytokine-targeted approaches to arthritis treatment.

Despite substantial therapeutic progress achieved through the development of disease-modifying antirheumatic drugs (DMARDs), maintaining effective and sustained control of RA remains challenging. A significant subset of patients fails to achieve adequate clinical improvement or experiences only partial therapeutic responses [16]. This continuing unmet need highlights the importance of exploring alternative mechanisms involved in RA pathogenesis, including immunological pathways shared with depression, and underscores the necessity of identifying novel therapeutic targets and developing more effective treatment strategies.

The aim of this study was to investigate whether sertraline influences functional performance independently of the extent of joint pathology in a rat model of collagen-induced arthritis (CIA), and to compare this relationship with that observed following standard anti-arthritic treatments, including methotrexate, infliximab, and tocilizumab, while examining associations between functional activity, histopathological changes, and selected systemic inflammatory markers.

## 2. Results

### 2.1. Body Weight

The final body weights differed from those recorded at the beginning of the experiment. Body weight changed over time across all groups, with increases noted in Groups A, D, F, and G, a slight rise in Group B, and reductions in Groups C and E. At the end of the experiment, significant intergroup differences were observed (*p* < 0.05), with the strongest contrasts between Groups A vs. C, A vs. E, and E vs. G (*p* = 0.000). Additional significant differences occurred for B vs. E (*p* = 0.007), C vs. D (*p* = 0.002), C vs. F (*p* = 0.005), D vs. E (*p* = 0.000), and E vs. F (*p* = 0.000). Detailed values are presented in Table 1.

### 2.2. Haematological Changes

Distinct alterations in blood cell parameters were observed across the experimental groups during the 12-week study period. All statistically significant differences (*p* < 0.05) are detailed in Table 2. In Group A, significant increases were noted in red blood cell count (RBC), haemoglobin concentration (HGB), and haematocrit (HCT), reflecting enhanced erythropoiesis. Group B showed a significant decrease in mean corpuscular volume (MCV) and an increase in mean corpuscular haemoglobin concentration (MCHC), indicating the emergence of smaller, more haemoglobin-dense erythrocytes. In Group C, haematological parameters at week 12 showed lower RBC, HGB, platelets (PLT), and neutrophil values compared with baseline. In contrast, Group D exhibited significant improvements in erythroid indices, accompanied by mild reductions in MCV and mean corpuscular haemoglobin (MCH), and increases in MCHC and red cell distribution width (RDW). Group E showed features consistent with altered erythroid parameters, characterized by macrocytosis, accompanied by decreases in RBC, HGB, HCT and neutrophil counts, as well as an increase in RDW. Group F experienced transient anaemia mid-study, with partial recovery by week 12, and significant changes in MCV, MCHC, and RDW. Finally, Group G displayed only modest haematological alterations, limited to MCV, MCHC, and RDW. All statistically significant differences (*p* < 0.05) are detailed in Table 2.

At the final time point (week 12), haematological parameters differed significantly between certain groups, reflecting the impact of treatments on blood profiles. Earlier time points showed no significant inter-group differences, so the focus is on end-point comparisons. The detailed haematological data are collected in Table 3.

### 2.3. Motor Activity

Wheel-running activity decreased significantly from baseline to week 12 in Groups B (*p* = 0.008), C (*p* = 0.008), D (*p* = 0.018), E (*p* = 0.008), and G (*p* = 0.008), whereas no significant changes were observed in Groups A and F. Among the groups showing a significant reduction, the sertraline-treated group exhibited a smaller numerical decline in exploratory wheel-running activity. After adjustment for baseline wheel-running activity and baseline body weight, no significant overall effect of treatment group on week-12 activity was detected [F(6,47) = 1.248, *p* = 0.3]. Although the adjusted mean activity was numerically higher in Group D than in Group B (40.78 vs. 14.70), the direct between-group contrast was not statistically significant [F(1,47) = 0.808, *p* = 0.373]. In the sensitivity analysis additionally adjusted for body weight change, neither the overall group effect [F(6,46) = 0.889, *p* = 0.511] nor the comparison between Groups D and B [F(1,46) = 0.746, *p* = 0.392] was statistically significant. Therefore, Group D was considered to show a smaller numerical decline in voluntary wheel-running activity and did not demonstrate preservation of functional performance.

### 2.4. Macroscopic Appearance

**Figure 1:**
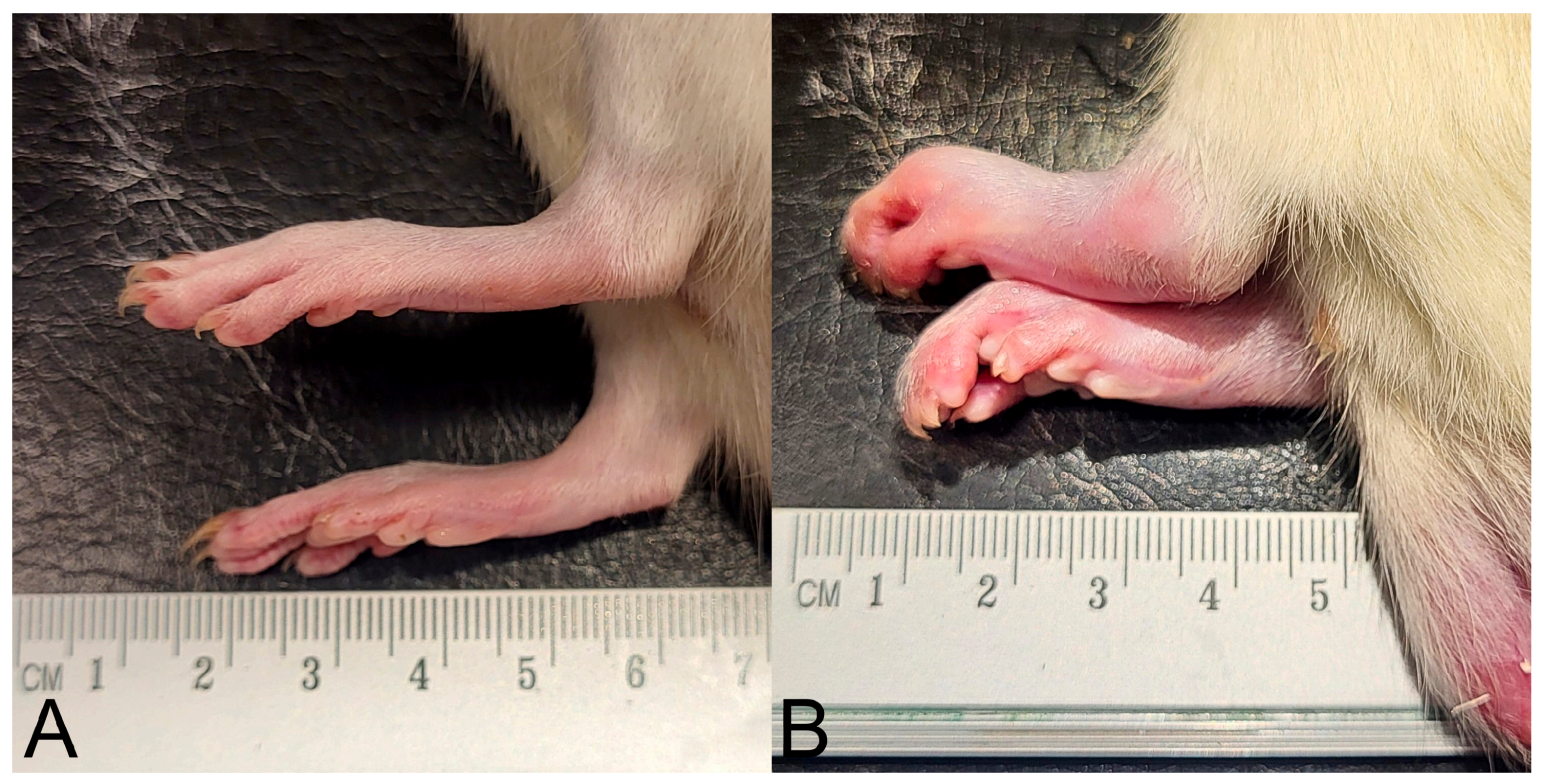
(**A**) Macroscopic appearance of the hind paws from a control rat (group A). (**B**) Macroscopic appearance of the hind paws from a rat with collagen-induced arthritis (group B).

The hind paws of Group A displayed a normal macroscopic appearance, whereas rats with collagen-induced arthritis (Group B) showed pronounced paw swelling indicative of inflammatory joint changes (Figure 1). Representative macroscopic images of the hind paw from treated groups (Group C–G) are provided in Supplementary Figures S1 and S2.

A statistically significant increase in the mean joint swelling index was observed in all experimental groups with induced arthritis compared to the control group. Groups B, D, and F showed significantly higher swelling indices than Group A (*p* = 0.000). Group C exhibited a significantly lower swelling index than Group B (*p* = 0.014). Group E also showed a reduced swelling index compared to Group B (*p* = 0.010) and to Group D (*p* = 0.041).

### 2.5. Histopathological Findings

**Figure 2:**
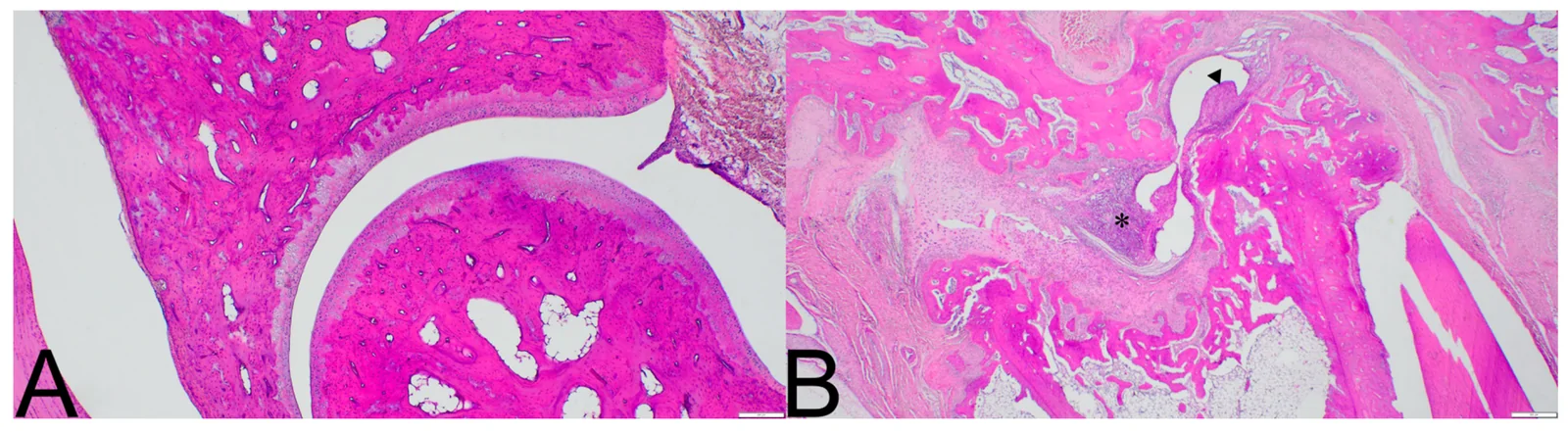
(**A**) Histologically no pathological changes were observed in control group (group A). Ankle joint, sagittal section, rat. H&E x40. Scale bar 200 µm. (**B**) Histological appearance of collagen-induced arthritis in a rat (group B). Synovial hyperplasia (arrowhead), severe inflammatory cells infiltration with pannus formation in the joint (asterisk), severe cartilage and bone erosion. Ankle joint, sagittal section, rat. H&E x40. Scale bar 200 µm.

**Figure 3:**
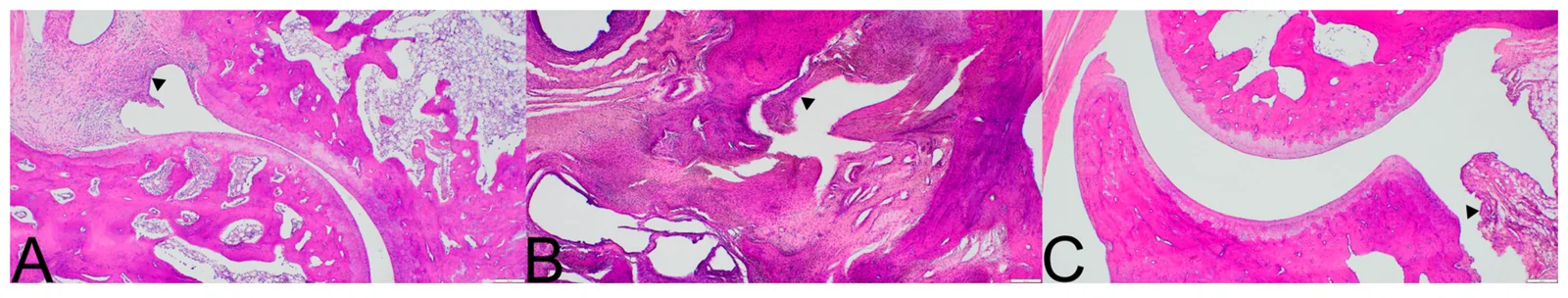
(**A**) Histological appearance of collagen-induced arthritis treated with methotrexate (group C). Synovial hyperplasia with moderate inflammatory cell infiltration (arrowhead). Ankle joint, sagittal section, rat. H&E x40. Scale bar 200 µm. (**B**) Histological appearance of collagen-induced arthritis treated with sertraline (group D). Synovial hyperplasia (arrowhead), severe cartilage and bone erosion. Ankle joint, sagittal section, rat. H&E x40. Scale bar 200 µm. (**C**) Histological appearance of collagen-induced arthritis treated with methotrexate and sertraline (group E). A few inflammatory cells infiltrating the synovium were observed (arrowhead). Ankle joint, sagittal section, rat. H&E x40. Scale bar 200 µm.

**Figure 4:**
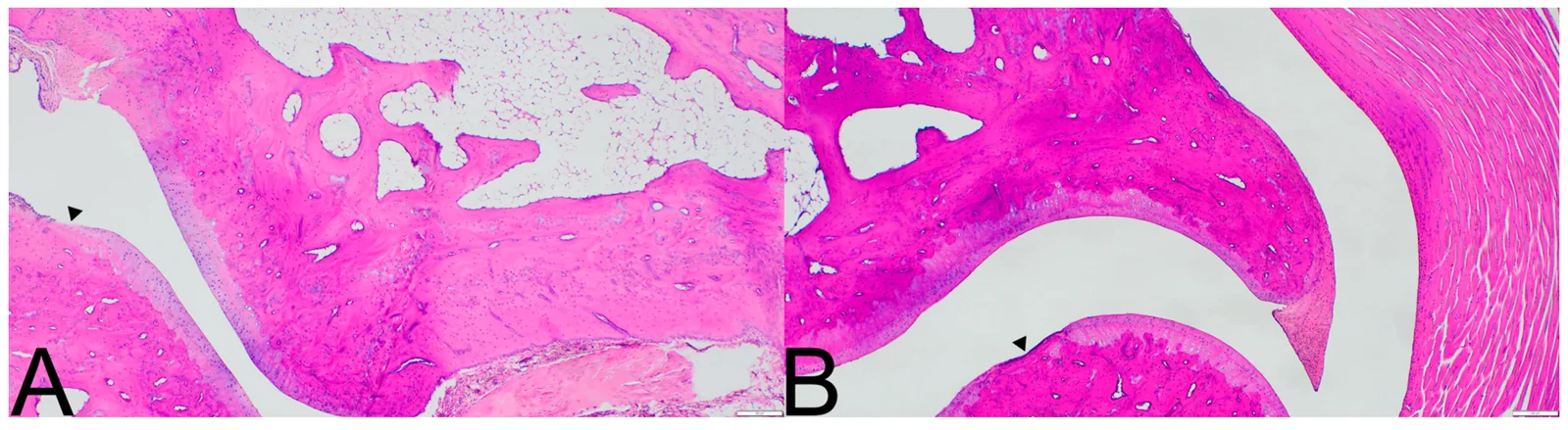
(**A**) Histological appearance of collagen-induced arthritis treated with infliximab (group F). (**B**) Histological appearance of collagen-induced arthritis treated with tocilizumab (group G). Reduced synovial hyperplasia, inflammatory infiltration, cartilage and bone erosion (arrowheads). Ankle joint, sagittal section, rat. H&E x40. Scale bar 200 µm.

Microscopic examination of the affected joints revealed significant, group-dependent differences in inflammation and tissue damage (Figure 2, Figure 3 and Figure 4).

The mean synovial inflammation index was significantly elevated in the experimental groups. Groups B and D had significantly higher synovial inflammation compared to Group A (*p* = 0.000). Group C showed a significantly lower inflammation index than Group B (*p* = 0.044). Group E differed significantly from both Group B (*p* = 0.001) and Group D (*p* = 0.003), while Group G also showed reduced inflammation compared to Group B (*p* = 0.021) and Group D (*p* = 0.042).

A statistically significant increase in synovial hyperplasia was observed in the experimental groups. Groups B and D showed significantly higher levels compared to Group A (*p* = 0.000 and *p* = 0.004, respectively). Group C differed significantly from Group B (*p* = 0.007), as did Group E (*p* = 0.008) and Group G (*p* = 0.046).

A significantly higher pannus formation index was recorded in the experimental groups compared to the control. Groups B and D had significantly elevated pannus formation compared to Group A (*p* = 0.000 and *p* = 0.005, respectively). Group C showed a significant reduction compared to Group B (*p* = 0.009). Group E also differed from Group B (*p* = 0.050), as did Group G (*p* = 0.034). Moreover, Group E showed significantly less pannus formation than Group D (*p* = 0.047).

Cellular inflammation was significantly elevated in groups with induced arthritis compared to the control. Groups B and D had significantly higher cellular inflammation than Group A (*p* = 0.000 and *p* = 0.001, respectively). Group C showed significantly lower values than Group B (*p* = 0.008). Group E differed significantly from Group B (*p* = 0.001) and Group D (*p* = 0.007). Group G showed a significantly lower index compared to Group B (*p* = 0.046). Group C also differed from Group D (*p* = 0.041).

A significantly increased mean bone erosion index was recorded in the experimental groups. Group B showed higher erosion compared to Group A (*p* = 0.001). Groups C, E, and G all showed significantly lower bone erosion compared to Group B (*p* = 0.014, *p* = 0.003, and *p* = 0.036, respectively).

Statistically significant increases in cartilage erosion were observed in the experimental groups. Groups B and D exhibited higher erosion than Group A (*p* = 0.001 and *p* = 0.011, respectively). Group C showed reduced cartilage erosion compared to Group B (*p* = 0.039), while Group E differed significantly from both Group B (*p* = 0.003) and Group D (*p* = 0.038).

Proteoglycan loss was significantly increased in all experimental groups compared to the control. Groups B, D, F, and G all exhibited significantly greater loss than Group A (*p* = 0.000, *p* = 0.032, *p* = 0.003, and *p* = 0.028, respectively). No significant differences were observed between group B and any of the treated groups (C, E, F, G) with regard to this parameter.

Group-level values for the joint swelling index and individual histopathological parameters expressed as median are summarized in Table 4.

**Figure 5:**
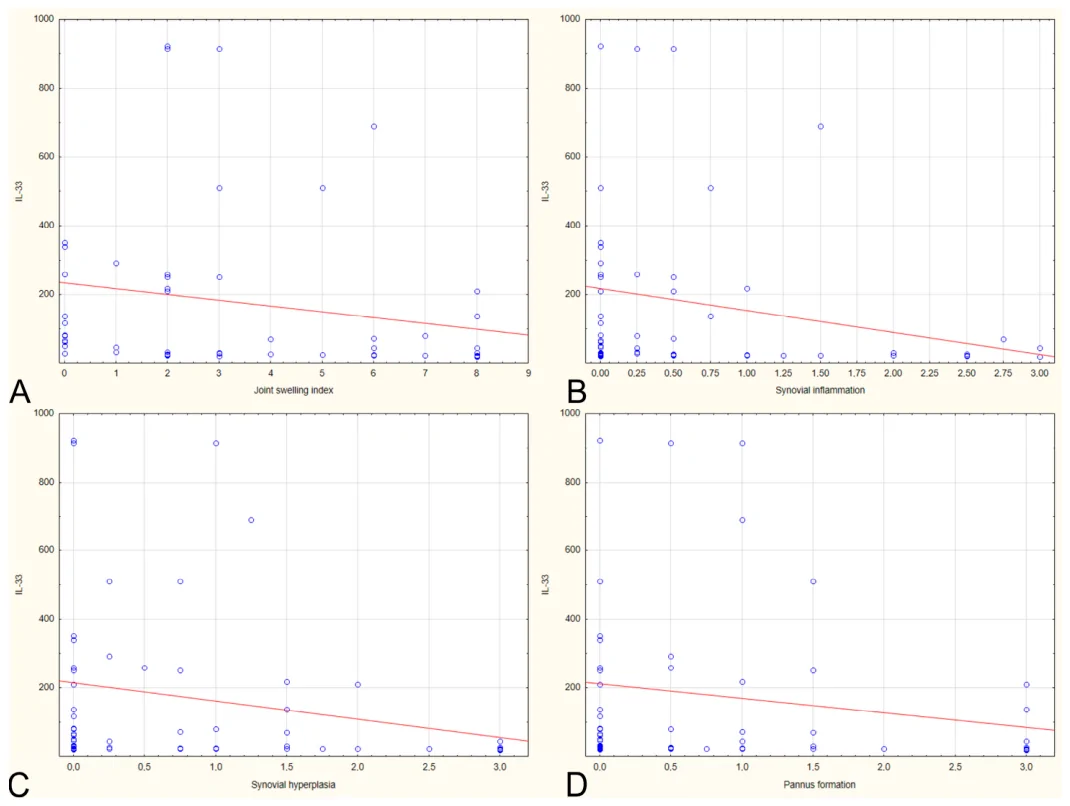
(**A**) A negative correlation between IL-33 concentration and the joint swelling index. IL-33 concentration decreased with increasing joint swelling index (r = −0.4; *p* = 0.003). (**B**) A negative correlation between IL-33 concentration and synovial inflammation. IL-33 concentration decreased with increasing synovial inflammation (r = −0.35, *p* = 0.008). (**C**) A negative correlation between IL-33 concentration and synovial hyperplasia (r = −0.31, *p* = 0.019). IL-33 concentration decreased with increasing synovial hyperplasia. (**D**) A negative correlation between IL-33 concentration and pannus formation. IL-33 concentration decreased with increasing pannus formation (r = −0.28, *p* = 0.035).

**Figure 6:**
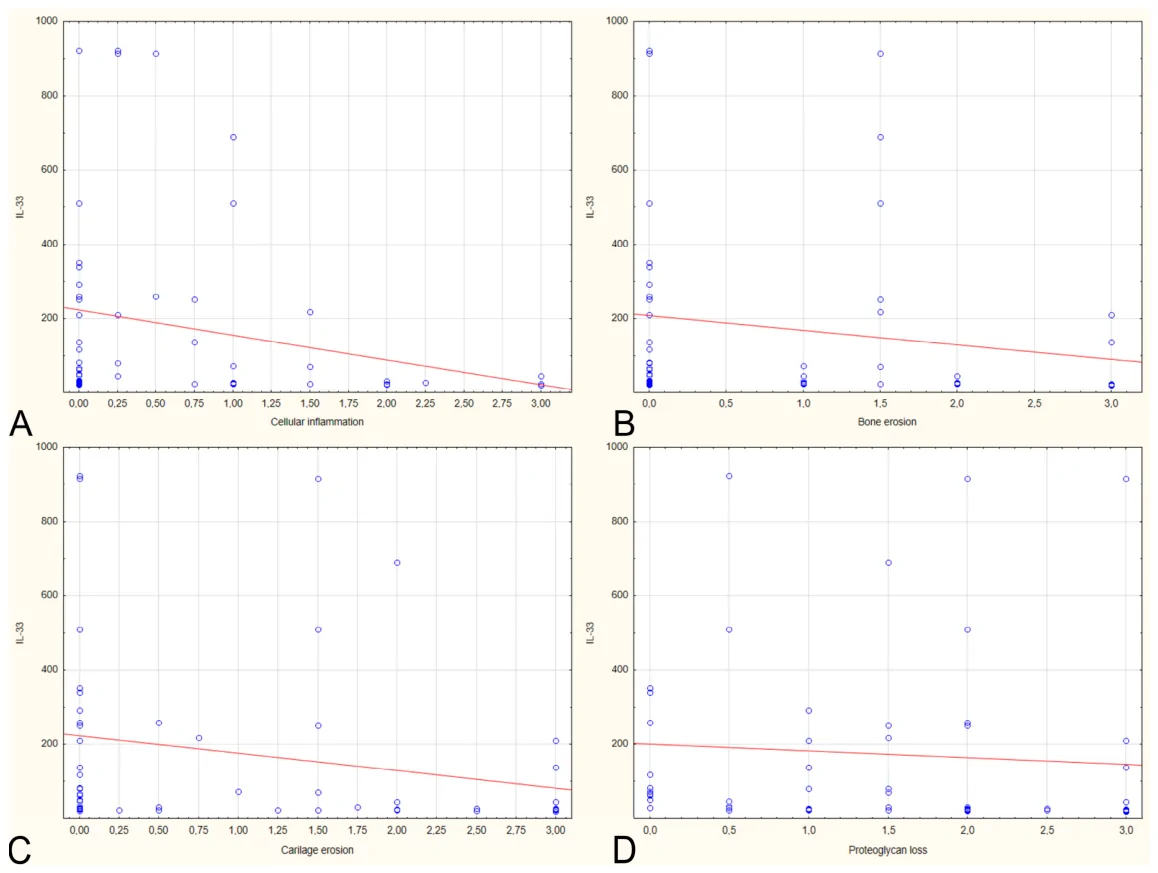
(**A**) A negative correlation between IL-33 concentration and cellular inflammation. IL-33 concentration decreased with increasing cellular inflammation (r = −0.37, *p* = 0.005). (**B**) A negative correlation between IL-33 concentration and bone erosion (r = −0.33, *p* = 0.012). (**C**) A negative correlation between IL-33 concentration and cartilage erosion. IL-33 concentration decreased with increasing cartilage erosion (r = −0.42, *p* = 0.001). (**D**) A negative correlation between IL-33 concentration and proteoglycan loss. IL-33 concentration decreased with increasing proteoglycan loss (r = −0.31, *p* = 0.02).

Correlation analysis revealed a strong positive association between mean oedema and multiple histopathological indices. These included the joint swelling index (r = 0.91, *p* = 0.002), synovial hyperplasia (r = 0.805, *p* = 0.016), bone erosion (r = 0.91, *p* = 0.002), and proteoglycan loss (r = 0.84, *p* = 0.009). A significant negative correlation was observed between IL-33 concentration and the joint swelling index (r = −0.4, *p* = 0.003) (Figure 5A). Lower IL-33 levels were associated with increased joint oedema. Similarly, IL-33 showed significant inverse correlations with several histopathological parameters, including synovial inflammation (r = −0.35, *p* = 0.008) (Figure 5B), synovial hyperplasia (r = −0.31, *p* = 0.019) (Figure 5C), pannus formation (r = −0.28, *p* = 0.035) (Figure 5D), and cellular inflammation (r = −0.37, *p* = 0.005) (Figure 6A). Negative correlations were also noted between IL-33 and indices of structural joint damage: bone erosion (r = −0.33, *p* = 0.012) (Figure 6B), cartilage erosion (r = −0.42, *p* = 0.001) (Figure 6C), and proteoglycan loss (r = −0.31, *p* = 0.02) (Figure 6D). No statistically significant differences were observed between experimental groups for TNF-α, IL-1β, or IL-6 concentrations.

## 3. Discussion

The aim of this study was to evaluate the effects of sertraline on joint pathology, haematological parameters, selected inflammatory cytokines, and exploratory measures of spontaneous motor activity in a rat model of collagen-induced arthritis, and to contextualize these findings in comparison with methotrexate, infliximab, and tocilizumab. Methotrexate, infliximab, and tocilizumab were included to represent conventional and targeted pharmacological approaches to the treatment of arthritis. The rationale for evaluating sertraline in the CIA model was based on evidence linking systemic inflammation with neuropsychiatric symptoms and on reports indicating that SSRIs may exert immunomodulatory effects. Pro-inflammatory cytokines, including TNF-α, IL-1β, and IL-6, have been implicated in depression-related neuroimmune alterations [17,18]. Sertraline has been reported to modulate inflammatory signalling in experimental and clinical settings, including reductions in IL-1β, TNF-α, CRP, and IL-6 [19,20,21]. These observations provided the basis for investigating whether sertraline could influence inflammatory and functional outcomes in the present CIA model.

The present findings reveal marked disparities in joint pathology among Groups A-G. In the untreated arthritis group, there was pronounced joint swelling accompanied by severe synovial inflammation, synovial hyperplasia, and pannus formation. This aggressive inflammatory pannus was associated with dense cellular infiltration in the joint and led to extensive bone and cartilage erosion with significant proteoglycan loss. By contrast, the intervention-treated Groups C, E, F, and G demonstrated markedly reduced pathological changes. These treated groups exhibited significantly less oedema and milder synovial inflammation, with minimal pannus formation relative to Group B. Correspondingly, cartilage destruction and bone erosion were substantially attenuated, and proteoglycan loss was limited in these cohorts. Group A showed no inflammation signs or tissue damage, as expected. These findings are consistent with previous reports showing that methotrexate attenuates joint inflammation in experimental arthritis models, although interpretation should consider potential systemic effects associated with the dosing regimen used [22,23]. The therapeutic effect of methotrexate has been attributed, at least in part, to its ability to suppress the expression of the pro-inflammatory cytokines such as TNF-α, IL-6 [24]. In the CIA model methotrexate not only alleviated joint inflammation and cartilage destruction but also reduced the expression of IL-1β, TNF-α, IL-6, and interleukin 17 (IL-17) in joint tissue and splenocyte cultures [25]. These effects should be interpreted with caution, as systemic changes observed in methotrexate-treated animals may have contributed to the apparent attenuation of disease parameters. Comparable anti-inflammatory effects have been reported with biologic agents. In a study conducted in monkeys, tocilizumab markedly reduced joint swelling, accompanied by a marked decrease in neutrophil infiltration in inflamed joints [26]. Likewise, infliximab treatment in CIA rats alleviated joint inflammation and effectively reduced paw oedema [27].

In our study, no statistically significant improvement in histopathological outcomes was detected with sertraline monotherapy compared with untreated arthritis. Joint swelling, synovial infiltration, and pannus formation remained pronounced in the sertraline treated group. These findings do not demonstrate histopathological equivalence between Groups B and D but indicate that sertraline did not produce a detectable improvement in the assessed structural outcomes.

Previous studies have reported inconsistent effects of sertraline and other SSRIs in experimental arthritis. Sertraline reduced clinical disease severity and TNF-α levels in adjuvant-induced arthritis, while fluoxetine and citalopram showed anti-inflammatory effects in collagen-induced arthritis and human rheumatoid arthritis synovial tissue [28,29]. In contrast, serotonergic modulation has also been associated with exacerbated inflammation in adjuvant arthritis, and high-dose sertraline increased acute carrageenan-induced paw oedema [30,31]. These contrasting findings suggest that the effects of SSRIs on inflammation may depend on the experimental model, dose, and timing of assessment. Such differences may contribute to the lack of structural joint protection observed with sertraline in the present CIA study. However, the present study did not directly assess central neurobiological mechanisms, and their potential contribution to the observed effects therefore remains uncertain.

In the present CIA model, wheel-running activity was interpreted as an exploratory composite behavioural outcome rather than specific measure of joint pain, mobility, or depressive-like behaviour. Compared with the adjuvant control group (Group A), untreated CIA rats (Group B) showed a marked decline in activity. Numerically higher activity was observed in rats treated with methotrexate (Group C), infliximab (Group F), or tocilizumab (Group G) than in untreated CIA rats. This pattern is directionally consistent with previous reports indicating that TNF-α inhibition and IL-6 receptor blockade may reduce inflammation-associated functional decline in CIA [27]. Wheel-running activity also decreased significantly from baseline in the sertraline-treated group (Group D), although a smaller numerical decline was observed than in untreated CIA rats (Group B). This descriptive finding does not establish that sertraline preserved functional performance or improved spontaneous activity independently of joint pathology. Wheel-running behaviour may be influenced by pain perception, fatigue, sickness behaviour, anxiety, motivation, body weight, and general physiological status [32].

Interpretation of wheel-running activity in inflammatory disease models requires caution, as this behaviour is influenced by multiple non-joint-specific factors, including sickness behaviour, sedation, anxiety, body weight changes, and overall physiological status. In the present study, methotrexate-treated animals exhibited reductions in body weight and alterations in haematological parameters, which may have influenced spontaneous locomotor activity independently of joint inflammation or pain. Accordingly, reduced wheel-running performance in methotrexate-treated groups should be interpreted with caution and cannot be considered a direct or isolated indicator of arthritis-related functional impairment. Depression, fatigue, and pain hypersensitivity are known to contribute to reduced physical activity in chronic inflammatory diseases [33], and experimental studies indicate that SSRIs may alter locomotor behaviour [34]. Therefore, the improvement in spontaneous activity observed in sertraline-treated rats may reflect enhanced well-being rather than the underlying inflammatory process. An important limitation of the present study is the absence of validated behavioural tests assessing depressive-like or anxiety-like phenotypes. Wheel-running activity cannot distinguish between changes related to depressive-like behaviour, pain, fatigue, sickness behaviour, anxiety, body weight, or general physiological condition. Therefore, the relative preservation of activity observed in sertraline-treated rats should not be interpreted as direct evidence of an antidepressant effect.

The body weight changes observed across the experimental groups likely reflected both chronic inflammation and the systemic effects of the administered treatments. Adjuvant control animals showed physiological weight gain, whereas untreated CIA rats gained little weight, consistent with the metabolic burden and catabolic state associated with chronic inflammation in RA [35,36]. Although methotrexate-treated animals in our study showed a reduction in body weight rather than stabilization or gain, this pattern is not universally reported. Zuo et al. demonstrated that MTX effectively restored physiological weight gain in CIA rats by counteracting inflammation-induced metabolic disturbances [37]. The different pattern observed in our study may reflect methotrexate-related gastrointestinal intolerance or reduced appetite, potentially compounded by the catabolic effects of chronic inflammation. Differences in MTX dose, route of administration, treatment frequency, disease severity, and strain-specific susceptibility may also have contributed to the discrepant findings.

By contrast, rats receiving sertraline alone displayed a weight trajectory closer to physiological growth. Although higher doses of sertraline have been shown to reduce appetite in experimental settings, the dose used in this study did not appear to exert a marked anorexigenic effect [38].

The most pronounced deviation from physiological weight gain was observed in the group receiving the combination of methotrexate and sertraline. The substantial reduction in body weight suggests a potential cumulative effect of both agents on appetite, gastrointestinal tolerance, or systemic energy balance. While methotrexate and sertraline are commonly used in clinical practice, little is known about their combined metabolic impact in the context of inflammatory disease, highlighting a need for further research into potential interactions between these therapies.

Biologic therapies produced a markedly different metabolic profile. Both infliximab and tocilizumab were associated with clear weight gain, suggesting that effective cytokine inhibition may reverse inflammation-driven metabolic disturbances. This observation aligns with evidence that suppression of TNF-α and IL-6 restores appetite regulation, reduces systemic catabolism, and normalizes neuroendocrine pathways affected by chronic inflammation [27]. However, it should be noted that human monoclonal antibodies were used as comparator treatments in a rat model; although infliximab and tocilizumab have been employed in experimental arthritis studies, species-specific differences in target engagement and immunogenicity cannot be excluded. Therefore, the outcomes observed in these groups should be interpreted as reference pharmacological effects rather than definitive evidence of selective TNF-α or IL-6 pathway inhibition.

Across the treated groups, clear differences in erythrocyte morphology were observed. Groups B, D and F showed smaller red blood cells with relatively elevated MCHC, which suggests microcytosis with haemoglobin-dense erythrocytes that could be associated with inflammatory stress and altered iron metabolism in chronic inflammatory conditions [39]. In contrast, Groups C and E demonstrated macrocytosis and anisocytosis, indicating disturbances in erythrocyte maturation, which may reflect impaired nucleic acid synthesis or increased turnover of erythroid precursors, mechanisms described in experimental models exposed to antifolate therapies and persistent inflammatory stimulation [40].

At the final time point, neutrophil counts in Group C were reduced compared with baseline values, and a similar pattern was noted in Group E. This finding is consistent with the immunomodulatory effects of methotrexate, which may influence leukocyte dynamics during long-term treatment [41]. Groups B, D and F showed a biphasic leukocyte response with an initial increase followed by later normalization, consistent with inflammatory activity diminishing over time [42]. Platelet dynamics also varied between groups. Group E showed an early rise in platelet counts followed by a decline at the endpoint, whereas Group C exhibited a more gradual reduction throughout the study period. Groups receiving biologic agents, including Groups F and G, maintained relatively stable haematological parameters.

In the present study, a significant negative correlation was observed between IL-33 concentration and both clinical and histopathological indices of joint inflammation and structural damage. Lower IL-33 levels were associated with higher joint swelling, more severe synovial inflammation, and greater destruction of articular cartilage and bone. These findings suggest that IL-33 may play a protective or regulatory role in the inflammatory cascade of CIA, potentially limiting the extent of tissue injury. Although IL-33 is commonly classified as a pro-inflammatory cytokine of the IL-1 family, its biological activity appears to be context-dependent [43]. Experimental studies have demonstrated that IL-33 can also exert anti-inflammatory effects through modulation of regulatory T cells (Tregs) and promotion of IL-10 secretion, thereby contributing to the resolution phase of inflammation [44]. The observed inverse relationships between IL-33 levels and markers of joint pathology in the present study are consistent with this concept of dual immunoregulatory action [45]. However, these associations are correlative in nature and do not establish a causal role of IL-33 in modulating joint pathology in the present study.

Interestingly, no statistically significant intergroup differences were found in the serum concentrations of TNF-α, IL-1β, or IL-6. Several methodological and biological factors may account for these findings. First, these cytokines are characterized by short plasma half-lives and rapid fluctuations during the inflammation [46], which may limit their detectability at single time points. Second, local (synovial) rather than systemic cytokine production may predominate in chronic arthritis, leading to weak correlations between serum levels and joint pathology [47]. Third, the timing of sample collection performed at the end of the 12-week experiment may have coincided with a phase of partial resolution, when systemic cytokine levels had already declined despite persistent histopathological lesions. Importantly, the absence of detectable differences in circulating cytokine levels in the present study does not contradict the established pathogenic role of TNF-α, IL-1β, and IL-6 in collagen-induced arthritis, which has been extensively demonstrated in experimental models using early time-course analyses, genetic deficiency approaches, and targeted cytokine blockade. Rather, these findings indicate that the cytokine measurements performed at the end of the experimental period do not provide sufficient resolution to support mechanistic interpretations regarding systemic inflammatory modulation. Finally, it is possible that compensatory anti-inflammatory mechanisms could have suppressed the systemic expression of classical pro-inflammatory mediators. Previous studies have consistently demonstrated increased IL-6 concentrations in CIA and shown that IL-6 deficiency or IL-6 receptor blockade markedly attenuates disease development, confirming the important role of this pathway in CIA [48,49,50]. Cytokine responses are time-dependent, with IL-6 increasing early after collagen immunization, whereas IL-1β and TNF-α become more prominent at later stages of inflammation [51].

The reduction in inflammatory mediators observed in sertraline-treated animals in other studies may provide a mechanistic link to these findings. Previous research demonstrated that sertraline significantly decreased the expression of key pro-inflammatory factors such as TNF-α, IL-1β, and inducible nitric oxide synthase (iNOS), and inhibited phosphorylation of NF-κB and its inhibitor IκB-α, thereby blocking activation of the NF-κB pathway [52]. Additionally, sertraline therapy decreased serum CRP concentrations [53], pro-inflammatory cytokines, including IL-1α, IL-6, IL-8, IL-12, and IFN-γ [54].

Several limitations of the present study should be acknowledged. First, the study was conducted exclusively in male rats to reduce biological variability. However, rheumatoid arthritis and RA-associated depression are more prevalent in women, and sex-related differences in immune responses, disease expression, and treatment effects have been documented in both clinical and experimental contexts [55]. Accordingly, the present findings may not fully generalize to females. Future studies including both sexes will be important to determine whether the observed effects of sertraline on functional performance and related outcomes are sex dependent. Second, validated assessments of depressive-like, anxiety-like, or pain-related behaviour were not performed. Wheel-running activity should therefore be interpreted as a composite measure of spontaneous activity influenced by pain, fatigue, sickness behaviour, motivation, body weight, and general physiological condition, rather than as a specific measure of depressive-like behaviour or joint function. Third, histopathological and serum cytokine assessments were performed only at the terminal time point, preventing evaluation of the temporal relationships between systemic inflammation, structural joint damage, and functional activity. Moreover, circulating cytokine concentrations may not accurately reflect local inflammatory processes within the joints. The absence of a completely naïve control group and the baseline differences in body weight between groups also limit the interpretation of treatment-related changes. Finally, infliximab and tocilizumab are human monoclonal antibodies, and species-specific differences in target engagement and immunogenicity cannot be excluded. Accordingly, the functional findings and the effects observed in the biologic-treatment groups should be interpreted cautiously.

## 4. Materials and Methods

### 4.1. Animals

Wistar rats Cmdb:Wi (male, 8 weeks old) were purchased from Centre for Experimental Medicine, Medical University of Białystok (Białystok, Poland). All rats were housed in cages under a 12 h light cycle at 25 ± 1 °C and 40–60% relative humidity and given tap water and commercially available food ad libitum. The present study was approved by the Local Ethical Committee for Animal Experiments in Olsztyn (decision No. 19/2024 from 20 March 2024). Experiments comply with the Act of 15 January 2015 on the Protection of Animals Used for Scientific or Educational Purposes (Journal of Laws 2015, item 266, Poland), which implements the provisions of Directive 2010/63/EU of the European Parliament and of the Council of 22 September 2010 on the protection of animals used for scientific purposes. Male rats were used to minimize variability related to ovarian hormone fluctuations; oestrogen status can modulate the incidence and severity of collagen-induced arthritis in rats [56]. The use of a single sex allowed for a more controlled assessment of treatment-related effects on functional performance and joint pathology.

### 4.2. Induction of Collagen-Induced Arthritis (CIA)

After a two-week acclimatization period, fifty-six male Wistar rats were allocated to seven experimental groups (*n* = 8 per group; Groups A–G) before collagen or vehicle administration. The allocation sequence was generated by simple randomization using a computer-generated random-number sequence. Randomization was not stratified according to body weight, and stratification according to arthritis severity was not applicable because group allocation preceded arthritis induction. Allocation was not formally concealed, and investigators responsible for arthritis induction and treatment administration were aware of group assignment. Arthritis severity and histopathological changes were assessed by two independent observers blinded to group allocation, whereas wheel-running activity was recorded automatically. Collagen-induced arthritis was induced according to the manufacturer’s recommendations for bovine type II collagen (Chondrex Inc., Woodinville, WA, USA) and in accordance with previously published protocols. Briefly, bovine type II collagen (2 mg/mL) dissolved in 0.05 M acetic acid was emulsified with an equal volume of incomplete Freund’s adjuvant (IFA) by gentle stirring using a hand-held homogenizer (1000–3000 rpm) in an ice-water bath to prevent overheating. Rats in Groups B–G received a subcutaneous injection of 200 μL of the collagen–IFA emulsion at the base of the tail. Group A received an equivalent volume of vehicle consisting of 0.05 M acetic acid emulsified with IFA and served as the adjuvant-injected control group. To enhance and synchronize arthritis development, a booster injection was administered on day 7 following the primary immunization, consisting of 100 μL of freshly prepared collagen–IFA emulsion injected subcutaneously at the tail base, as described previously [48,57].

### 4.3. Treatment

Two weeks after inflammation induction, rats were randomly divided into seven experimental groups:

Group A (adjuvant control): vehicle (0.05 M acetic acid + IFA), no collagen, no treatment.

Group B positive control (CIA): rats with CIA receiving no pharmacological treatment.

Group C (CIA + MTX): rats with CIA treated with methotrexate (MTX, Accord Healthcare, Warsaw Poland) (5 mg/kg, intraperitoneally, once weekly).

Group D (CIA + SRT): rats with CIA treated with sertraline (SRT KRKA, Nove mesto, Slovenia) (10 mg/kg, orally, once daily).

Group E (CIA + MTX + SRT): rats with CIA treated with a combination of methotrexate (5 mg/kg i.p., once weekly) and sertraline (10 mg/kg p.o., once daily).

Group F (CIA + IFX): rats with collagen-induced arthritis treated with infliximab (IFX; Remicade, Janssen-Cilag International NV, Beerse, Belgium) at a dose of 5 mg/kg, administered intraperitoneally once weekly starting at week 2 after arthritis induction. This dosing regimen and route of administration were selected based on previous experimental studies in rat models of collagen-induced arthritis in which infliximab was used as a pharmacological comparator to inhibit TNF-α-mediated inflammation [27,58].

Group G (CIA + TCZ): rats with collagen-induced arthritis treated with tocilizumab (TCZ; RoActemra, Roche Pharma AG, Grenzach-Wyhlen, Germany) at a dose of 8 mg/kg, administered intraperitoneally every two weeks starting at week 2 after arthritis induction. This dosing regimen and route of administration were selected based on previous experimental studies employing IL-6 receptor blockade in animal models of collagen-induced arthritis [59].

### 4.4. Functional Assessment

Functional assessment included longitudinal measurements of spontaneous locomotor activity using the wheel-running test, as well as body weight monitoring and joint swelling evaluation. These parameters were used as indicators of joint function, disease-associated disability, and overall functional performance.

### 4.5. Evaluation of Arthritis

After arthritis induction, the size of hind paws was measured using Vernier callipers, body weight was recorded, and the arthritis index of the hind limbs was evaluated at regular intervals—weeks 0, 4, 8, 12 (for clarity, week 0 was defined as the day of collagen-induced arthritis induction, prior to the development of clinical signs and before initiation of any pharmacological treatment). CIA severity was evaluated using a standardized scoring method. Each hind limb was individually assessed and graded as follows: 0—no abnormalities; 1—redness and mild swelling limited to the tarsal or ankle region; 2—redness and mild swelling spreading from the ankle to the midfoot; 3—redness and moderate swelling extending from the ankle to the metatarsal area; 4—redness and pronounced swelling involving the ankle, entire foot, and toes. Arthritis severity was evaluated using a scoring system ranging from 0 to 4, with the cumulative score of both hind paws serving as the arthritis index, with a maximum value of 8. All clinical assessments were performed independently by two experienced observers who were blinded to the experimental groups and were unaware of the treatment allocation of the animals. In cases of discrepant scores, a consensus value was established following joint re-evaluation. This clinical scoring system (0–4) was used exclusively for macroscopic assessment of joint swelling and was independent of the histopathological scoring, which was performed separately using a 0–3 scale in accordance with SMASH recommendations [59].

### 4.6. Motor Activity

Motor activity was initially assessed using a running wheel apparatus, allowing rats to familiarize themselves with the device before the experimental procedures began. After this habituation phase, locomotor performance was quantified by recording the number of wheel rotations completed during a fixed period. Measurements were obtained at two time points: at baseline prior to the onset of clinical arthritis, corresponding to the day of collagen immunization (week 0; T1), and at the end of the study (week 12; T3), providing an index of changes in motor activity throughout the experiment. Wheel-running activity was used as an exploratory functional endpoint reflecting overall spontaneous activity rather than a specific measure of joint pain or structural impairment. This parameter is known to be influenced by multiple factors, including sickness behaviour, body weight changes, fatigue, and general well-being [60].

### 4.7. Euthanasia Procedure

At the end of the treatment period (week 12–T3), rats were anesthetised by intraperitoneal administration of a ketamine (150 mg/kg; Vetketam, Vet-Agro, Lublin, Poland) and xylazine (20 mg/kg; Sedazin, Biowet, Puławy, Poland) solution. Following induction of deep anaesthesia animals were then humanely euthanized by cervical dislocation with transection of the spinal cord, performed by a trained operator while the animals remained fully anesthetised. This method is permitted for laboratory rodents weighing less than 1 kg and subjected to prior sedation or anaesthesia, in accordance with Annex IV of Directive 2010/63/EU on the protection of animals used for scientific purposes.

### 4.8. Histological Analysis

For histopathological assessment, the rats were euthanized after 12 weeks and their secondary ankle joints were collected. The joints were fixed in buffered 10% formalin, decalcified using Osteodec (BioOptica, Milan, Italy), and subsequently embedded in paraffin wax, cut, stained with Mayer’s haematoxylin and eosin (HE) and toluidine blue. The slides were evaluated under a light microscope (BX63, Olympus, Tokyo, Japan) using cellSense (cellSens Standard Version 4.2 CS-ST-V4.2, Olympus, Tokyo, Japan) software. Arthritis severity was assessed in a blinded manner using a semi-quantitative 0–3 scoring system in accordance with the SMASH recommendations for standardized microscopic arthritis scoring of histological sections from inflammatory arthritis animal models. Histopathological evaluation was performed on paraffin-embedded sections of hind paws, the recommended anatomical site for systemic inflammatory arthritis models such as collagen-induced arthritis. Distinct pathological features were evaluated as separate parameters, including synovial membrane hyperplasia and inflammatory cell infiltration, pannus formation, cartilage damage, bone erosion, and loss of proteoglycan content, using a 0–3 grading scale reflecting increasing severity based on predefined morphological criteria derived from established SMASH definitions. For each animal, at least two non-serial sections obtained at comparable cutting planes were analysed, and multiple ankle and tarsal joints were evaluated per section to account for local heterogeneity of joint involvement. All histological assessments were performed independently by two experienced observers who were blinded to experimental group allocation, and final scores were established by consensus following joint re-evaluation in cases of discrepant assessments [59,61]. The use of the SMASH framework provides a validated and widely accepted reference standard for histopathological assessment in experimental inflammatory arthritis, ensuring reproducibility and comparability with other CIA studies.

### 4.9. Haematological Analysis

Blood samples were collected at baseline on the day of CIA induction (week 0; T1), after 4 weeks (week 4; T2), and after 12 weeks (week 12; T3), haematological analysis was performed immediately after sampling. The analysis was performed using a ProCyte Dx haematology analyser (IDEXX Laboratories, Westbrook, MA, USA).

The concentrations of inflammatory cytokines TNF-alfa, IL-1β, IL-6, interleukin 33 (IL-33) in the serum of CIA rats were analysed with enzyme-linked immunosorbent assay (Thermo Fisher Scientific, Waltham, MA, USA) according to the manufacturer’s instruction.

### 4.10. Statistical Analysis

The variables were checked for normality using the Shapiro–Wilk test. Differences in body weights, haematological parameters, selected cytokine levels, and histopathological parameters between groups were analysed using the Kruskal–Wallis H test (one-way ANOVA on ranks) followed by Dunn’s post hoc test. Additionally, those parameters at different points of time were analysed using the Wilcoxon paired test. Correlations between histopathological parameters, haematological parameters, selected cytokine levels, and arthritis index were examined using Spearman’s rank correlation (r: Spearman’s rank correlation coefficient); *p*  ≤  0.05 was considered statistically significant, and *p*  ≤  0.001 was considered highly significant. An additional baseline-adjusted analysis of covariance (ANCOVA) was performed for week-12 wheel-running activity, with treatment group as the categorical factor and baseline wheel-running activity and baseline body weight as covariates. Homogeneity of regression slopes was assessed by testing the group-by-baseline activity and group-by-baseline body weight interactions. A direct contrast was used to compare the sertraline-treated group (Group D) with the untreated CIA group (Group B). As a sensitivity analysis, body weight change from baseline to week 12 was additionally included as a covariate. The statistical analysis was performed using Statistica 14 software (StatSoft Inc., Tulsa, OK, USA).

## 5. Conclusions

In the present study, no statistically significant improvement in histopathological outcomes was detected with sertraline monotherapy compared with untreated CIA. Although the sertraline-treated group showed a smaller numerical decline in voluntary wheel-running activity than the untreated CIA group, the adjusted between-group comparison was not statistically significant. Accordingly, the present findings do not demonstrate that sertraline preserves functional performance independently of joint pathology. The observed activity pattern should be regarded as exploratory and cannot be attributed to antidepressant, analgesic, or centrally mediated mechanisms on the basis of the present data. Further studies using larger cohorts and validated assessments of locomotor function, pain, fatigue, and affective behaviour are needed to determine whether sertraline influences non-articular determinants of activity in inflammatory arthritis.

## Figures and Tables

**Table 1:** Mean body weight values recorded at three time points: before induction of CIA (T1), after 4 weeks (T2), and after 12 weeks (T3).

Group	Mean Body Weight T1	Mean Body Weight T2	Mean Body Weight T3	Δ Weight T3-T1
A	347.63 ± 26.66	382.13 ± 19.22	392.75 ± 17.13	45.13
B	398.88 ± 16.63	386.0 ± 42.82	403.75 ± 37.47	4.88
C	437.88 ± 4.32	412.63 ± 27.85	420.0 ± 22.67	−17.88
D	396.75 ± 39.42	399.63 ± 70.98	413.13 ± 70.43	16.38
E	420.88 ± 5.17	392.38 ± 23.21	378.25 ± 26.90	−42.63
F	415.25 ± 11.26	405.13 ± 29.64	431.88 ± 22.07	16.63
G	465.25 ± 25.82	456.38 ± 48.49	484.0 ± 47.45	18.75

**Table 2:** Statistically significant changes in haematological parameters from baseline to week 12 in experimental groups (A–G). Values are mean ± SD at baseline and at week 12 for each group, * *p* < 0.05.

Parameter	Group A	Group B	Group C	Group D	Group E	Group F	Group G
RBC (M/µL)	9.08 ± 2.43 → 10.93 ± 0.11 *	9.10 ± 1.38 → 9.44 ± 1.51	10.56 ± 1.73 → 7.79 ± 1.86 *	9.23 ± 1.25 → 10.96 ± 0.37 *	9.52 ± 0.34 → 7.33 ± 0.6 *	10.16 ± 0.49 → 9.65 ± 0.31	9.71 ± 0.63 → 9.49 ± 0.33
HCT (%)	48.88 ± 3.34 → 53.6 ± 0.67 *	46.01 ± 6.25 → 45.08 ± 6.79	49.48 ± 1.62 → 40.25 ± 9.25	45.98 ± 5.83 → 52.51 ± 1.54 *	47.18 ± 1.99 → 38.42 ± 2.2 *	51.08 ± 2.3 → 46.33 ± 1.81	48.54 ± 2.62 → 45.99 ± 1.45
HGB (g/dL)	15.6 ± 1.1 → 17.5 ± 0.2 *	14.55 ± 2.03 → 14.79 ± 1.95	15.55 ± 0.94 → 13.18 ± 2.87	14.76 ± 1.9 → 17.15 ± 0.58 *	15.20 ± 0.5 → 12.56 ± 0.79 *	16.35 ± 0.85 → 15.30 ± 0.58 *	15.74 ± 1.04 → 15.33 ± 0.51
MCV (fL)	49.54 ± 0.77 → 49.2 ± 0.79	51.99 ± 4.7 → 48.53 ± 1.12 *	50.86 ± 1.14 → 51.78 ± 2.27	49.9 ± 1.15 → 47.94 ± 0.52 *	49.16 ± 1.11 → 52.56 ± 2.09 *	50.24 ± 0.99 → 48.00 ± 0.59	49.9 ± 1.45 → 48.5 ± 1.62 *
MCH (pg)	15.91 ± 0.27 → 16.1 ± 0.13	16.09 ± 0.53 → 15.71 ± 0.66	15.93 ± 0.3 → 16.96 ± 0.55	16.01 ± 0.29 → 15.64 ± 0.18 *	15.93 ± 0.38 → 17.18 ± 0.48	16.03 ± 0.21 → 15.85 ± 0.23	16.11 ± 1.45 → 16.15 ± 0.53
MCHC (g/dL)	30.08 ± 0.69 → 32.44 ± 0.24	31.56 ± 0.51 → 32.89 ± 0.79 *	31.89 ± 0.64 → 32.81 ± 0.79	32.12 ± 0.33 → 32.66 ± 0.28 *	32.24 ± 0.33 → 32.69 ± 0.71	31.90 ± 0.45 → 33.02 ± 0.43 *	32.33 ± 0.64 → 33.31 ± 0.36 *
RDW (%)	22.48 ± 2.47 → 24.44 ± 0.68	22.51 ± 1.47 → 24.59 ± 2.79	23.09 ± 0.52 → 28.90 ± 2.06	22.28 ± 2.09 → 25.98 ± 0.64 *	22.76 ± 0.73 → 29.8 ± 2.82 *	23.39 ± 0.72 → 24.25 ± 1.19 *	21.64 ± 7.03 → 23.76 ± 1 *
WBC (K/µL)	5.37 ± 1.55 → 4.32 ± 0.37	4.26 ± 0.69 → 4.11 ± 1.03	4.85 ± 0.79 → 3.19 ± 0.93	5.01 ± 1.54 → 4.15 ± 0.64	4.64 ± 0.5 → 3.47 ± 1.06 *	5.27 ± 0.91 → 4.79 ± 0.9	5.03 ± 0.93 → 4.36 ± 1.38
Neutrophils (K/µL)	1.36 ± 0.5 → 1.34 ± 0.25	1.40 ± 0.44 → 0.92 ± 0.42	1.28 ± 0.44 → 0.06 ± 0.08	1.12 ± 0.36 → 1.00 ± 0.32	1.1 ± 0.17 → 0.031 ± 0.02 *	1.41 ± 0.57 → 1.16 ± 0.35	1.49 ± 0.7 → 1.27 ± 0.8
PLT (K/µL)	493 ± 247 → 641 ± 59	626 ± 92 → 679 ± 261	615 ± 92 → 407 ± 237	682 ± 49 → 673 ± 135	648 ± 32 → 327 ± 148 *	657 ± 62 → 748 ± 129	588 ± 27 → 729 ± 107

**Table 3:** Statistically significant intergroup differences in haematological parameters.

Parameter	Group Comparison	*p*-Value
RBC	A vs. C	0.001
	A vs. E	0.000
	C vs. D	0.003
	D vs. E	0.000
HCT	A vs. C	0.001
	A vs. E	0.000
	C vs. D	0.006
	D vs. E	0.000
HGB	A vs. C	0.001
	A vs. E	0.000
	C vs. D	0.005
	D vs. E	0.000
MCV	B vs. C	0.000
	B vs. E	0.001
	C vs. D	0.010
	C vs. F	0.012
	D vs. E	0.003
	E vs. F	0.003
MCH	B vs. C	0.015
	B vs. E	0.003
	C vs. D	0.001
	C vs. E	0.037
	C vs. F	0.0037
	D vs. E	0.007
	E vs. F	0.007
RDW	A vs. C	0.017
	A vs. E	0.010
	B vs. C	0.022
	B vs. E	0.013
	C vs. F	0.007
	C vs. G	0.001
	E vs. F	0.004
	E vs. G	0.001
Neutrophils	A vs. C	0.000
	A vs. E	0.000
	C vs. F	0.020
	C vs. G	0.015
	E vs. F	0.020
PLT	B vs. E	0.033
	C vs. F	0.023
	E vs. F	0.001
	E vs. G	0.008

**Table 4:** Swelling Index and histopathological assessment of arthritis severity presented as median (Q1–Q3) across the experimental groups.

Parameter	Group A	Group B	Group C	Group D	Group E	Group F	Group G
Swelling Index	0 (0–0)	7.5 (6–8)	2 (1–2.25)	6.5 (4.75–8)	2 (0.75–2.25)	4 (2–7.25)	2.5 (2–4.5)
Synovial inflammation	0 (0–0)	1.75 (1.5–2.125)	0.125 (0–0.4375)	2.25 (0.875–2.5625)	0 (0–0.0625)	0.5 (0.25–0.5625)	0.125 (0–0.3125)
Synovial hyperplasia	0 (0–0)	2.25 (1.4375–3)	0 (0–0.1875)	1.5 (0.75–2.0625)	0 (0–0.25)	0.75 (0.375–1.125)	0.25 (0–0.25)
Pannus formation	0 (0–0)	3 (1.75–3)	0 (0–0.25)	1.5 (1–1.875)	0 (0–0.125)	1 (0.5–1.875)	0.25 (0–0.5)
Cellular infiltration	0 (0–0)	2 (1.75–3)	0 (0–0.25)	1.5 (1–2.0625)	0 (0–0.0625)	0.5 (0.25–0.75)	0.125 (0–0.8125)
Bone erosion	0 (0–0)	2.5 (2–3)	0 (0–0.375)	1 (1–1.625)	0 (0–0)	0.75 (0–1.875)	0 (0–1)
Cartilage erosion	0 (0–0)	2.25 (2–3)	0.125 (0–0.5625)	1.875 (1.5–2.25)	0 (0–0)	1 (0–1.875)	0.25 (0–2.125)
Proteoglycan loss	0 (0–0)	2.5 (1.875–3)	1.5 (0.5–1.625)	1.75 (0.875–2.5)	1 (0.875–1.25)	2 (1.5–2.25)	1.5 (0.875–2.25)

## Data Availability

The datasets generated and analysed during the current study are available from the corresponding author upon reasonable request.

## Supplementary Materials

The following supporting information can be downloaded at: https://www.mdpi.com/article/10.3390/ijms27167251/s1.

## Abbreviations

The following abbreviations are used in this manuscript:

RA	Rheumatoid arthritis
TNF-α	Tumour necrosis factor-α
IL-6	Interleukin-6
IL-1β	Interleukin-1β
CRP	C-reactive protein
NMDA	N-methyl-D-aspartate
SRT	Sertraline
SSRI	Selective serotonin reuptake inhibitor
IL-10	Interleukin-10
DMARDs	Disease-modifying antirheumatic drugs
CIA	Collagen-induced arthritis
MTX	Methotrexate
INF	Infliximab
TCZ	Tocilizumab
HE	Haematoxylin and eosin
IL-33	Interleukin-33
RBC	Red blood cell count
HGB	Haemoglobin
HCT	Haematocrit
MCV	Mean corpuscular volume
MCHC	Mean corpuscular haemoglobin concentration
PLT	Platelets
MCH	Mean corpuscular haemoglobin
RDW	Red cell distribution width
5-HT	Serotonin
NE	Norepinephrine
IL-17	Interleukin-17
IL-1α	Interleukin-1 alpha
IL-8	Interleukin-8
IL-12	Interleukin-12
iNOS	Inducible nitric oxide synthase
IκB-α	Inhibitor of nuclear factor kappa B alpha
NF-κB	Nuclear factor kappa B
